# Lycium barbarum polysaccharides modulate SOCS-1 gene methylation and promote the maturation and migration of human monocyte-derived dendritic cells *in vitro*

**DOI:** 10.3389/fimmu.2026.1921722

**Published:** 2026-10-01

**Authors:** Wei Pan, Lijia Zhang, Yin Chen, Yuanjun Jiao, Huili Gu, Jiayan Gao, Lixin Jiang, Gaochao Qian

**Affiliations:** 1 Division of Education and Research, Changzhou Traditional Chinese Medicine Hospital Affiliated to Nanjing University of Chinese Medicine, Changzhou, China; 2 Department of General Medicine, Health Center of Nanjing Audit University, Nanjing, China; 3 Department of Hepatobiliary and Pancreatic Surgery, Peking University Shenzhen Hospital, Shenzhen, China; 4 Department of Clinical Laboratory, Changzhou West Taihu Hospital, Changzhou, China

**Keywords:** chemotaxis, dendritic cells, Lycium barbarum polysaccharides, methylation, SOCS-1

## Abstract

**Objective:**

To investigate the effects of *Lycium barbarum* polysaccharides (LBPs) on the methylation of the Suppressor of Cytokine Signalling-1 (SOCS-1) gene and the maturation and migration of human monocyte-derived dendritic cells (DCs) *in vitro*.

**Methods:**

Human monocyte-derived DCs were generated using recombinant human GM-CSF and interleukin-4 (IL-4) and treated with various concentrations of LBPs (50, 100, and 200 mg/L). SOCS-1 methylation and relative gene expression were analyzed by methylation-specific quantitative PCR (qMSP) and quantitative real-time PCR, respectively. DC surface markers were assessed by flow cytometry. Cytokine (IL-12 and IL-10) secretion was measured by enzyme-linked immunosorbent assay (ELISA). JAK2, STAT3 and STAT5 phosphorylation were assessed by Western Blot Analysis. The ability of LBPs-treated DCs to stimulate lymphocyte proliferation was evaluated in a mixed lymphocyte reaction (MLR). DC migration was tested using a Transwell assay toward MIP-3β, and CCR7 expression was quantified by real-time PCR.

**Results:**

LBPs treatment upregulated SOCS-1 gene methylation and increased the expression of maturation markers (CD80, CD83, HLA-DR) and IL-12 production in a dose-dependent manner. Conversely, SOCS-1 expression and IL-10 secretion were downregulated. Furthermore, LBPs-treated DCs enhanced allogeneic lymphocyte proliferation. A significant increase in DC migration toward MIP-3β and CCR7 expression was observed in the 200 mg/L LBPs group compared to the control.

**Conclusion:**

These findings indicate that LBPs are associated with increased SOCS-1 methylation and enhanced maturation and migratory capacity of human monocyte-derived DCs *in vitro*. Notably, to our knowledge, this is the first report demonstrating that a plant-derived polysaccharide can modulate SOCS-1 gene methylation, suggesting a potential epigenetic mechanism underlying the immunomodulatory activity of LBPs.

## Introduction

Dendritic cells (DCs) are the most potent antigen-presenting cells (APCs), playing pivotal roles in initiating and regulating immune responses. Their unique ability to activate diverse immune cells, including T cells, NK cells, and B cells, is crucial for pathogen clearance and inflammatory responses (1). Accumulating evidence indicates that the generation and function of DCs are often impaired within the tumor microenvironment, underscoring their critical role in anti-tumor immunity (2). Consequently, DC-based cancer vaccines have emerged as a promising immunotherapeutic strategy (3). The efficacy of such vaccines largely depends on DC maturation, a process essential for the migration of antigen-loaded DCs to secondary lymphoid organs and subsequent T cell priming. Current research focuses on enhancing DC maturation, co-stimulatory molecule expression, and antigen presentation to overcome tolerance to tumor-associated antigens (4). Numerous studies and clinical trials have demonstrated the potential of DC vaccines to induce anti-tumor immune responses and clinical benefits in cancers such as Non-small cell lung cancer (NSCLC) (5)and melanoma (6).

Modulating key regulatory genes and chemokine receptors presents a viable approach to control DC maturation and migration. For instance, suppressor of cytokine signaling-1 (SOCS-1) is a critical negative regulator of antigen presentation in DCs (7). Silencing SOCS-1 has been shown to enhance DC maturation, increase secretion of pro-inflammatory cytokines like IL-12, and potentiate antigen-specific cytotoxic T lymphocyte (CTL) responses and anti-tumor activity (8, 9). Additionally, the CC chemokine receptor 7 (CCR7) is a master regulator of DC migration toward lymph nodes, where they interact with T cells (10). Its expression is upregulated upon maturation, and its signaling is essential for directed chemotaxis (11, 12). Therefore, investigating factors that regulate SOCS-1 and CCR7 is crucial for understanding DC functional maturation.

Traditional Chinese medicine (TCM) has shown potential in modulating epigenetic mechanisms, such as DNA methylation, offering a novel avenue for regulating gene expression in immune cells (13). *Lycium barbarum* L. (goji berry) is a widely used medicinal plant, and its polysaccharides (LBPs) are considered major bioactive components responsible for various pharmacological effects, including antioxidant, immunomodulatory, and anti-cancer activities (14–18). Previous studies indicate that LBPs can promote the phenotypic and functional maturation of murine bone marrow-derived DCs (19). However, their effects on human monocyte-derived DCs, particularly concerning SOCS-1 methylation and migratory capacity, remain unclear.

This study aimed to investigate the effects of LBPs on human monocyte-derived DCs. We examined the modulation of SOCS-1 gene methylation, expression of maturation markers, cytokine profile, T cell stimulatory capacity, and migratory function via the CCR7-MIP-3β axis.

## Materials and methods

### Materials and agents

Peripheral blood samples were provided by Changzhou Blood Center (Changzhou, China). Peripheral blood mononuclear cells were isolated within 2 h after blood donation (n=8, age range 25-45, 4 male and 4 female, all healthy non-smokers; For each experiment, cells were isolated from individual donors, and data are presented as the mean ± SD from these independent biological replicates). LBPs for clinical application were purchased from Pharmagenesis Inc. (Newtown, PA, USA). We have performed a comprehensive characterization of the LBPs used in this study. Endotoxin level was less than 0.03 EU/mL by the quantitative Limulus Amebocyte Lysate (LAL) assay; The purity of LBPs was evaluated to be 85.21% by HPGPC (20); The weight-average molecular weight (Mw) was determined to be 3.2 kDa by high-performance gel permeation chromatography (HPGPC) (21); The monosaccharide composition analysis, performed by pre-column derivatization high-performance liquid chromatography (HPLC/UHPLC), revealed that LBPs is typically composed of glucose, galactose, arabinose, rhamnose, xylose, mannose, as well as uronic acids including galacturonic acid and glucuronic acid (22). Among these, neutral sugars (glucose, galactose, and arabinose) accounted for 91.5% of the total monosaccharide molar ratio; The Congo red assay indicated the presence of a triple-helix conformation; The Protein and polyphenol content, quantitatively determined by the Bradford method, were found to be less than 5%;This fully characterizes the material and meets the quality control standards expected for natural product studies. The study was carried out with the approval of the Ethic Committee of Changzhou traditional Chinese medicine hospital affiliated Nanjing University of Traditional Chinese Medicine. Written informed consent was obtained from each subject.

RPMI-160 culture medium, fetal bovine serum (FBS), and double antibody (penicillin-streptomycin) were obtained from Gibco (Grand Island, New York, USA). Lymphocyte isolation liquid was purchased from Axis Shield (Oslo, Norway). Real Time PCR kit was obtained from Invitrogen Life Technologies Corporation (Carlsbad, CA, USA). CD83-PE, HLA-DR-PE, and CD80-FITC, CD86-FITC, CD11c-PE and CCR7-APC antibodies were obtained from BD Biosciences (San Jose, CA). RevertAid™ First Strand cDNA Synthesis Kit was obtained from Fermentas (Vilnius, Lithuania). Quantitative PCR Kit was obtained from Hoffmann-La Roche Inc.(SYBR Green qPCR Master Mix, Nutley, NJ).

### The culture and LBPs-induced maturation of DCs

Peripheral blood mononuclear cells (MNCs) were obtained by Ficoll density gradient centrifugation. Cells were plated at the concentration of 5×10^6^cells/mL in RPMI 1640 medium supplemented with 10% FBS, and cultured in flask at 37 °C with 5% CO_2_ in a humidified atmosphere for 2 h. Subsequently, non-adherent cells were washed with phosphate buffer solution (PBS), while the adherent monocytes were cultured in RPMI-1640 complete medium in 5000 U/mL recombinant human (rh) GM-CSF and 2000 U/mL rhIL-4 for 6 days. On d3, half of the medium was renewed with fresh RPMI-1640 complete medium supplemented with rhGM-CSF and rhIL-4. On d6, immature DCs were harvested and seeded in 24-well plates at the density of 1×10^6^/mL with additional adding 200 µg/L LPS to obtain mature DCs. In the meantime, LBPs at different concentrations (50, 100, 200 mg/L) were added. Cells with no interference of LBPs served as control. Afterwards, DCs were collected after 4 h induction for Western Blot Analysis and 24 h induction for the other further study. In the anti-IL-10 neutralizing antibody experiment, mature DCs were treated with the anti-IL-10 neutralizing antibody (15 mg/L; R&D Systems, clone 25209) or isotype control for 48 h at 37 °C in 5% CO_2_, after which cells were harvested for CCR7 expression analysis and Cell migration of DCs. Untreated immature DCs, LPS-only treatment, and LBPs-only treatment were included as control conditions for subsequent experiments.

### Bisulfite conversion and methylation-specific quantitative PCR (qMSP) for SOCS1 methylation

qMSP were performed to assess the methylation status of the SOCS1 promoter CpG island. Genomic DNA extraction was performed with commercial kit according to the manufacturer’s instructions. Sample (2 μg) was bisulfite-treated using the EpiTect Bisulfite Kit (Qiagen). Methylation-specific primers targeting the 5’UTR region of SOCS1 were used: M-F: GAGAATTCGAAGGATTTGTCGG and M-R: ATTCCGAAACTACTATCGCGCA for methylated alleles; U-F: GAAATTTAAAGAGAATTTGAAGGATTTGTT and U-R: AAAATTCCAAAACTACTATCACACA for unmethylated alleles. Real-time PCR was performed with SYBR Green Master Mix, using bisulfite-converted DNA as template. Cycling conditions included initial denaturation at 95 °C for 10 min, followed by 45 cycles of 95 °C for 15 s and 60 °C for 1 min, with a melt-curve step for specificity verification. Fully methylated and unmethylated DNA standards were used as controls. Relative methylation levels were calculated via the 2^-ΔΔCt^ method or standard-curve quantification. All reactions were run in triplicate. Methylation levels were expressed as Beta value (alu which was calculated as the ratio of the methylated signal to the sum of the methylated and unmethylated signals: β = 2^(Ct-U - Ct-M)/[1 + 2^(Ct-U - Ct-M)], where Ct-M and Ct-U represent the threshold cycle values for methylated and unmethylated alleles, respectively. This calculation assumes equal amplification efficiency for both primer sets, which was verified by standard curve analysis (efficiency > 95% for both sets).

### 5-Aza rescue experiments

Rescue experiments were performed using the DNA methyltransferase inhibitor 5-aza-2′-deoxycytidine (5-Aza,Sigma). Mature DCs were cultured with LBPs (200 mg/L) for 48 h, followed by co-incubation with 5-Aza at different concentrations (10μmol/L,20μmol/L,50μmol/Land 100μmol/L) for an additional 72 h. Cells treated with LBPs alone, 5-Aza alone, or vehicle control were included as controls. After treatment, cells were harvested and assessed for SOCS-1 promoter methylation via methylation-specific PCR and expression of SOCS-1 gene.

### Real-time quantitative PCR

Total RNA was extracted from the mature DCs using Trizol reagent according to the manufacturer’s instructions. The cDNA synthesis was carried out with approximately 2 μg RNA using the RevertAid™ First Strand cDNA Synthesis Kit. Real-time PCR amplification was performed using SYBR Green I dye (Molecular Probes, Inc, Oregon, USA) on a MagNA Pure LC System with the primers listed in **Table 1**. PCR reactions were performed in a total volume of 20 μL containing 10 μL 2×SYBRPremix, 1 µL each specific primer to a final concentration of 10 μmol/L, and 1 µL cDNA template. The amplification reactions were as follows: pre-denaturation at 95 °C for 10 min, followed by 45 cycles of denaturation at 95 °C for 15 s, annealing at 60 °C for 40 s, and extension at 72 °C for 20 s. The β-actin was used as reference gene. The amplification results for real-time PCR was calculated as 2(-ΔΔCt), according to the previous description (23).

**Table 1:** Specific primers of target gene.

Gene	Forward primer	Reverse primer
SOCS-1	5’-AACTGCTTTTTCGCCCTTAGC-3’	5’-CAGCTCGAAGAGGCAGTCG -3’
CCR7	5’- GACCGATACCTACCTGCTCAAC-3’	5’- AAAGTGGACACCGAAGACCC-3’
DNMT3A	5’-TATTGATGAGCGCACAAGAGAGC-3’	5’- GGGTGTTCCAGGGTAACATTGAC-3’
DNMT3B	5’- GGCAAGTTCTCCGAGGTCTCTG-3’	5’- TGGTACATGGCTTTTCGATAGGA-3’
β-actin	5’- AGTTGCGACACCCTTTCTTG-3’	5’-GCTGTCACCTTCACCGTTCC-3’

### Identification of surface marker of mature DCs

Surface marker expression on mature DCs was analyzed by flow cytometry (BD FACSCalibur; Becton-Dickinson, Franklin Lakes, NJ, USA). After 7 days of culture, DCs were harvested, washed twice with PBS containing 1% bovine serum albumin (BSA), and adjusted to a density of 1 × 10^6^ cells/mL. Aliquots of 100 µL cell suspension (1 × 10^5^ cells) were dispensed into flow cytometry tubes and stained with the following fluorescein-labeled monoclonal antibodies at 4 °C for 30 min in the dark: phycoerythrin (PE)-conjugated anti-human CD83 (clone HB15e), PE-conjugated anti-human HLA-DR (clone L243), fluorescein isothiocyanate (FITC)-conjugated anti-human CD80 (clone L307.4), FITC-conjugated anti-human CD86 (clone 2331 FUN-1), PE-conjugated anti-human CD11c (clone B-ly6), and allophycocyanin (APC)-conjugated anti-human CCR7 (clone 150503). All antibodies were purchased from BD Biosciences. Appropriate mouse isotype-matched IgGs (BD Biosciences) served as negative controls. Following incubation, cells were washed twice with PBS-BSA, fixed with 1% paraformaldehyde, and analyzed using a flow cytometer. For each sample, at least 10,000 events were acquired within the gated cell population based on forward scatter (FSC) and side scatter (SSC) parameters. Data were processed using CellQuest Pro software (BD Biosciences), and the mean fluorescence intensity (MFI) as well as the percentage of positive cells were recorded for each marker.

### ELISA assay

IL-10 and IL-12p70 in the supernatants of mature DCs were analyzed using quantitative ELISA kits purchased from R&D Systems Inc (Minneapolis, Minnesota, USA), according to the manufacturer’s instructions. The absorbance was measured at 420 nm using a microplate reader (Wellscan MK3, Labsystems Dragon, Finland).

### Mixed lymphocyte reaction

After 7 days of culture, mature DCs were harvested and treated with mitomycin C (25 mg/L) at 37 °C for 30 min in the dark to arrest proliferation. The cells were then washed three times with PBS and resuspended in complete RPMI 1640 medium. Allogeneic non-adherent lymphocytes, serving as responder cells, were labeled with carboxyfluorescein diacetate succinimidyl ester (CFSE) at a final concentration of 2.5 µmol/L at 37 °C for 15 min. The labeling reaction was terminated by the addition of ice-cold RPMI 1640 medium containing 10% FBS, followed by two washes. Stimulator DCs and responder lymphocytes were then co-cultured in 96-well U-bottom plates at a stimulator-to-responder ratio of 1:10 (200 µL/well). Responder cells cultured alone served as the negative control. After 96 h of incubation at 37 °C in a 5% CO_2_ atmosphere, cells were collected and analyzed by flow cytometry. For flow cytometric analysis, lymphocytes were gated based on forward scatter (FSC) and side scatter (SSC) parameters to exclude debris and dead cells. CFSE fluorescence was detected in the FL1 channel (530 nm) following 488 nm excitation. A minimum of 10,000 events within the lymphocyte gate were acquired for each sample. The acquired data were analyzed using ModFit™ software, which deconvolutes CFSE fluorescence histograms into successive generation peaks. The proliferation index (PI) was calculated as the total number of divisions divided by the number of cells that entered the division process, and the stimulation index (SI) was determined as the ratio of PI in the co-cultured group to that in the control group.

### Cell migration of DCs

DC migration was assessed using a Transwell system (8 µm pore size; Corning, MA, USA) as previously described (24). Inserts were equilibrated in serum-free RPMI 1640 at 37 °C for 1 h prior to use. Mature DCs were harvested, washed, and resuspended in serum-free RPMI 1640 at 1 × 10^6^ cells/mL. Cells (5 × 10^5^ in 500 µL) were seeded into the upper chamber, while the lower compartment contained 600 µL of serum-free RPMI 1640 supplemented with or without rhMIP-3β (200 µg/L) as the chemoattractant or negative control, respectively. After 4 h of incubation at 37 °C in 5% CO_2_, cells that had migrated into the lower chamber were collected, pelleted (300 g, 5 min), and stained with APC-conjugated anti-human CD11c antibody (clone B-ly6; BD Biosciences) at 4 °C for 20 min in the dark. Following staining, cells were washed and resuspended in PBS for flow cytometric analysis. CD11c^+^ cells were quantified by gating on the lymphocyte/monocyte scatter profile, with a minimum of 10,000 events acquired per sample. The migration index was calculated as the ratio of CD11c^+^ cell counts in the rhMIP-3β-treated group to those in the control group. DCs were pre-incubated with an anti-CCR7 blocking antibody (10 mg/L; R&D Systems, clone 150503) or isotype as control for 1 h at 37 °C, followed by a Transwell migration assay toward MIP-3β.

### Western blot analysis

Protein samples were diluted to equal concentrations, boiled at 100 °C for 5 min, and separated on 10% SDS-PAGE gels using a Mini-PROTEAN^®^ Tetra Cell system (Bio-Rad, Hercules, CA, USA). Gels were prepared with 30% Acrylamide/Bis Solution (Bio-Rad) and polymerized with 10% ammonium persulfate and TEMED (Sigma-Aldrich, St. Louis, MO, USA). After electrophoresis at 120 V using Tris-Glycine SDS Running Buffer (1×, 25 mM Tris, 192 mM glycine, 0.1% SDS; Bio-Rad), proteins were transferred onto PVDF membranes (Immobilon^®^-P, Merck Millipore, Darmstadt, Germany) using a Trans-Blot^®^ Turbo™ Transfer System (Bio-Rad) at 350 mA for 1 h. Membranes were blocked with 5% non-fat milk (Bio-Rad) in TBST (20 mM Tris, 150 mM NaCl, 0.1% Tween-20, pH 7.6) for 1 h at room temperature, then incubated with primary antibodies (1:2000, Cell Signaling Technology, Danvers, MA, USA) overnight at 4 °C. After three TBST washes, membranes were incubated with HRP-conjugated secondary antibodies (Anti-rabbit HRP-conjugated secondary antibody, Catalog #7074; Anti-mouse HRP-conjugated secondary antibody, Catalog #7076;1:1000, Cell Signaling Technology) for 1 h at room temperature. Protein bands were visualized using enhanced chemiluminescence (ECL; Immobilon^®^ Western Chemiluminescent HRP Substrate, Merck Millipore) and exposed to X-ray film (Fujifilm, Tokyo, Japan). Band intensities were quantified using ImageJ software (NIH, Bethesda, MD, USA), and relative expression levels were calculated as the ratio of target protein to β-actin internal control.

### Statistical analysis

Data are presented as mean ± standard deviation (SD). Statistical analyses were performed using SPSS 17. software. All data were analyzed by one-way analysis of variance (ANOVA), followed by Tukey’s honestly significant difference (HSD) *post-hoc* test for multiple comparisons. A p-value <0.05 was considered statistically significant.

## Results

### Modulation of DNMT3A, DNMT3B, and SOCS-1 methylation and expression in dendritic cells by LBPs

Methylation-specific quantitative PCR (qMSP) revealed that SOCS-1 Beta value (β) displayed slight methylation in DCs treated with 50 mg/L LBPs, with markedly increased methylation levels at 100 mg/L and 200 mg/L (**Figure 1A**). Correspondingly, SOCS-1 mRNA expression was downregulated in a dose-dependent manner, exhibiting significant reductions at 100 mg/L (*p < 0.05) and 200 mg/L (**p < 0.01) relative to the control group (**Figure 1B**). In the 5-Aza rescue experiments, cell viability of primary dendritic cells (DCs) was evaluated using the CCK-8 assay after treatment with 5-Aza at concentrations of 0, 10, 20, 50, and 100 μmol/L for 72 h. Relative to the untreated control group, cell viability was 98.2 ± 1.8% (10 μM), 96.4 ± 2.1% (20 μM), 94.7 ± 2.9% (50 μM), and 91.3 ± 3.5% (100 μM), respectively. Furthermore, the rescue experiments demonstrated that 5-Aza treatment effectively reversed LBPs-induced SOCS-1 hypermethylation (**Figure 1C**) and concomitantly restored SOCS-1 expression (**Figure 1D**). Additionally, LBPs treatment significantly upregulated the mRNA expression of DNMT3A (**Figure 1E**) and DNMT3B (**Figure 1F**), the upstream epigenetic enzymes, in a dose-dependent manner.

**Figure 1:**
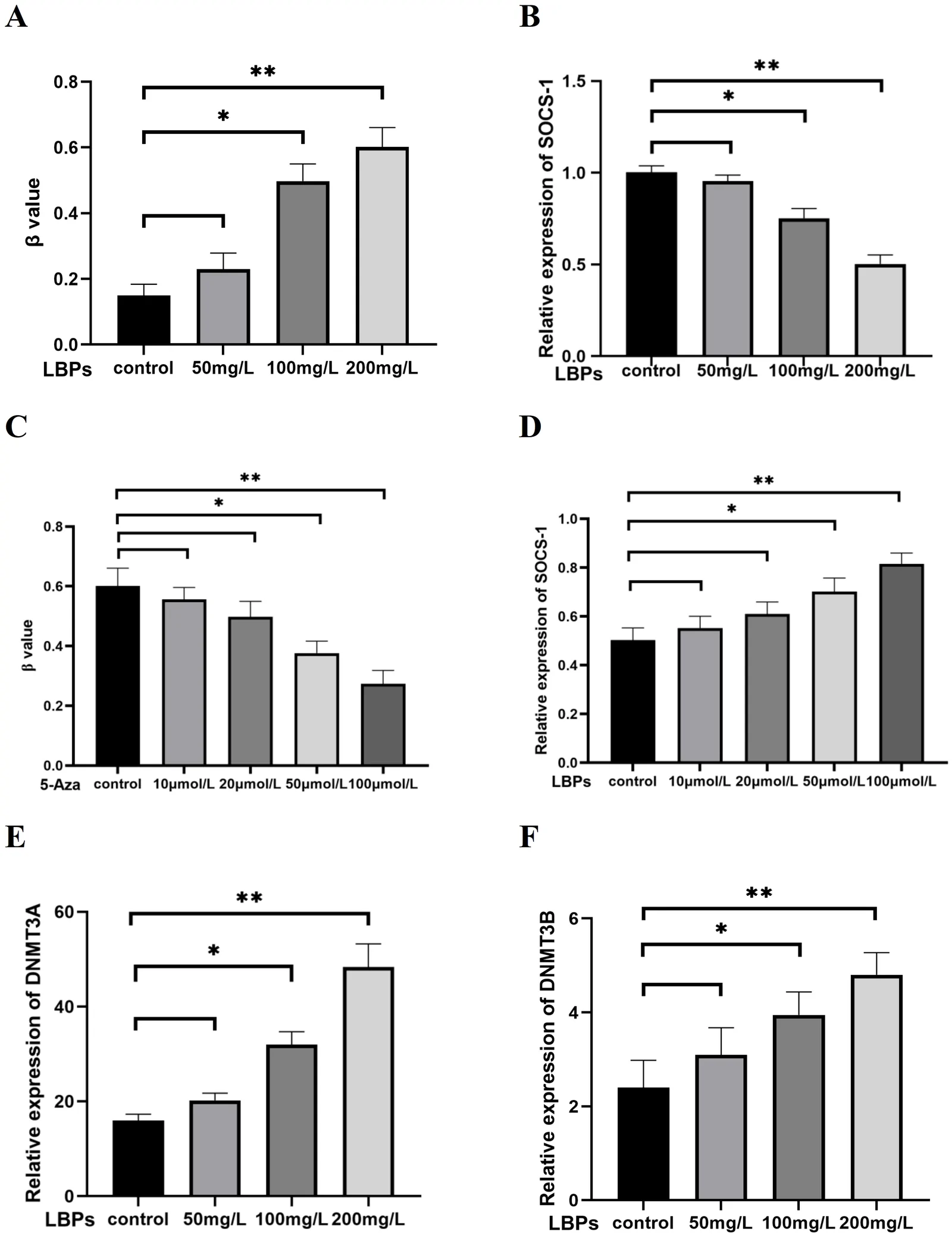
Effects of LBPs on DNA methylation and gene expression in dendritic cells. **(A)** methylation-specific quantitative PCR (qMSP) of SOCS-1 promoter in DCs treated with increasing concentrations of LBPs (0, 50, 100, and 200 mg/L). **(B)** SOCS-1 mRNA expression levels in DCs under the same LBPs treatment conditions. **(C)** Reversal of LBPs-induced SOCS-1 hypermethylation by 5-Aza treatment. **(D)** Restoration of SOCS-1 expression by 5-Aza treatment in LBPs-treated DCs. **(E, F)** mRNA expression levels of DNMT3A **(E)** and DNMT3B **(F)** in DCs following LBPs treatment at various concentrations. All data are shown as mean ± SD (n = 8 independent biological replicates). *p < 0.05, **p < 0.01 compared with the control group.

### LBPs enhance JAK/STAT signaling activation, promote DC maturation, and stimulate pro-inflammatory cytokine production

Western blot analysis revealed that LBPs treatment significantly enhanced the phosphorylation levels of JAK2, STAT3, and STAT5 in a dose-dependent manner (**Figure 2A**). Concurrently, LBPs treatment modulated cytokine secretion in DCs: IL-12 production was markedly elevated at 100 mg/L and 200 mg/L (P < 0.01), but not at 50 mg/L (P > 0.05), whereas IL-10 production was significantly suppressed at 100 mg/L (P < 0.05) and 200 mg/L (P < 0.01) compared with the control group (**Figure 2B**). In parallel, flow cytometric analysis demonstrated that LBPs treatment dose-dependently increased the surface expression of DC maturation markers, including CD80, CD83, CD86, and HLA-DR. Specifically, the expression levels of CD80, CD83, and HLA-DR in the 200 mg/L LBPs group were significantly higher than those in the control group (**Figure 2C**).

**Figure 2:**
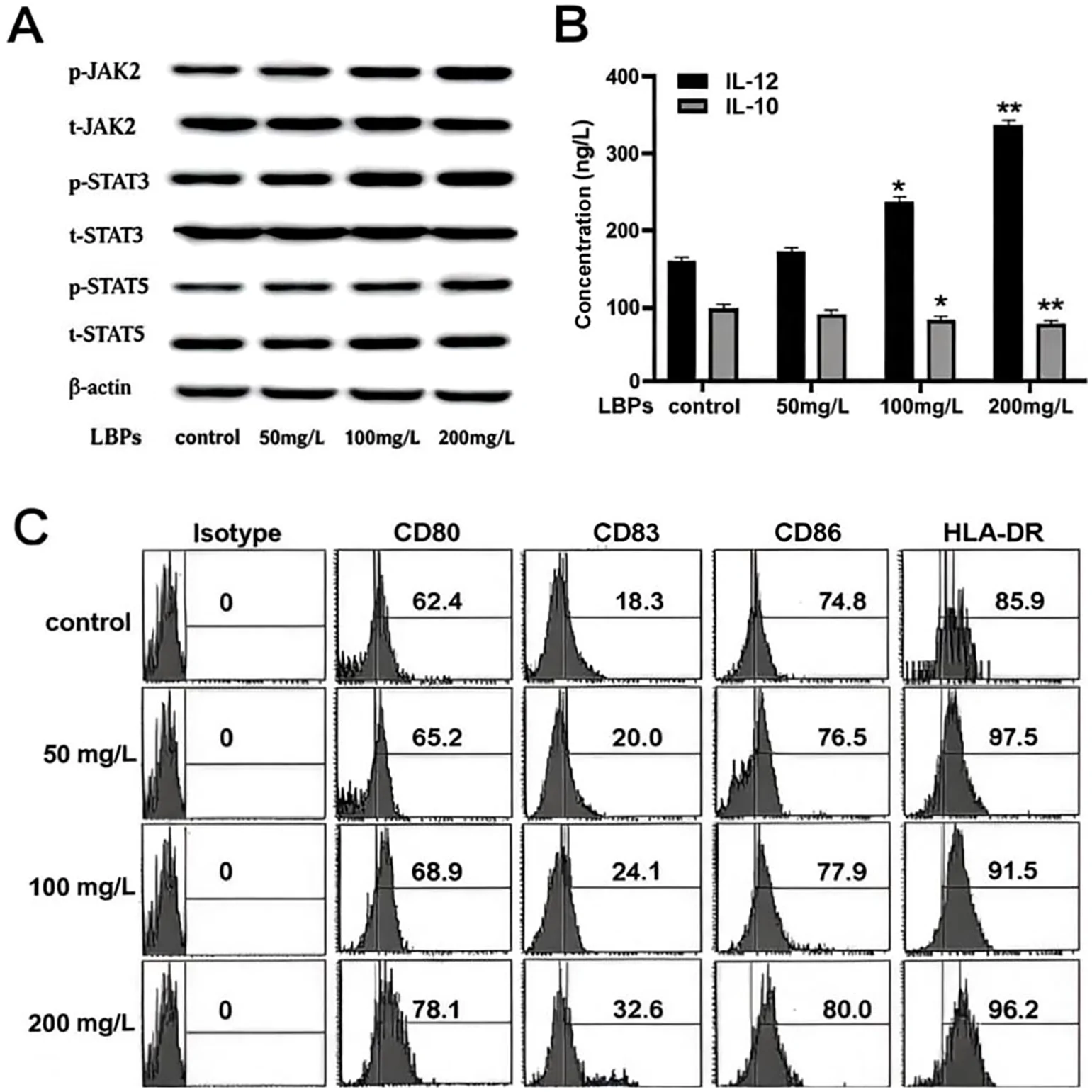
LBPs inhibit JAK/STAT signaling and promote DC maturation with a pro-inflammatory cytokine profile. **(A)** Western blot analysis demonstrated that LBPs treatment significantly enhanced the phosphorylation levels of JAK2, STAT3, and STAT5 without affecting their total protein expression. **(B)** LBPs treatment markedly elevated IL-12 production and suppressed IL-10 production at 100 mg/L and 200 mg/L compared with the control group. **(C)** LBPs dose-dependently upregulated the surface expression of DC maturation markers, including CD80, CD83, CD86, and HLA-DR. All data are shown as mean ± SD (n = 8 independent biological replicates). *p < 0.05, **p < 0.01 compared with the control group.

### LBPs-treated DCs enhance lymphocyte proliferation

DCs treated with higher concentrations of LBPs (100 and 200 mg/L) significantly potentiated the proliferation of allogeneic T lymphocytes in the MLR (p < 0.01, **Figure 3**; **Table 2**).

**Figure 3:**
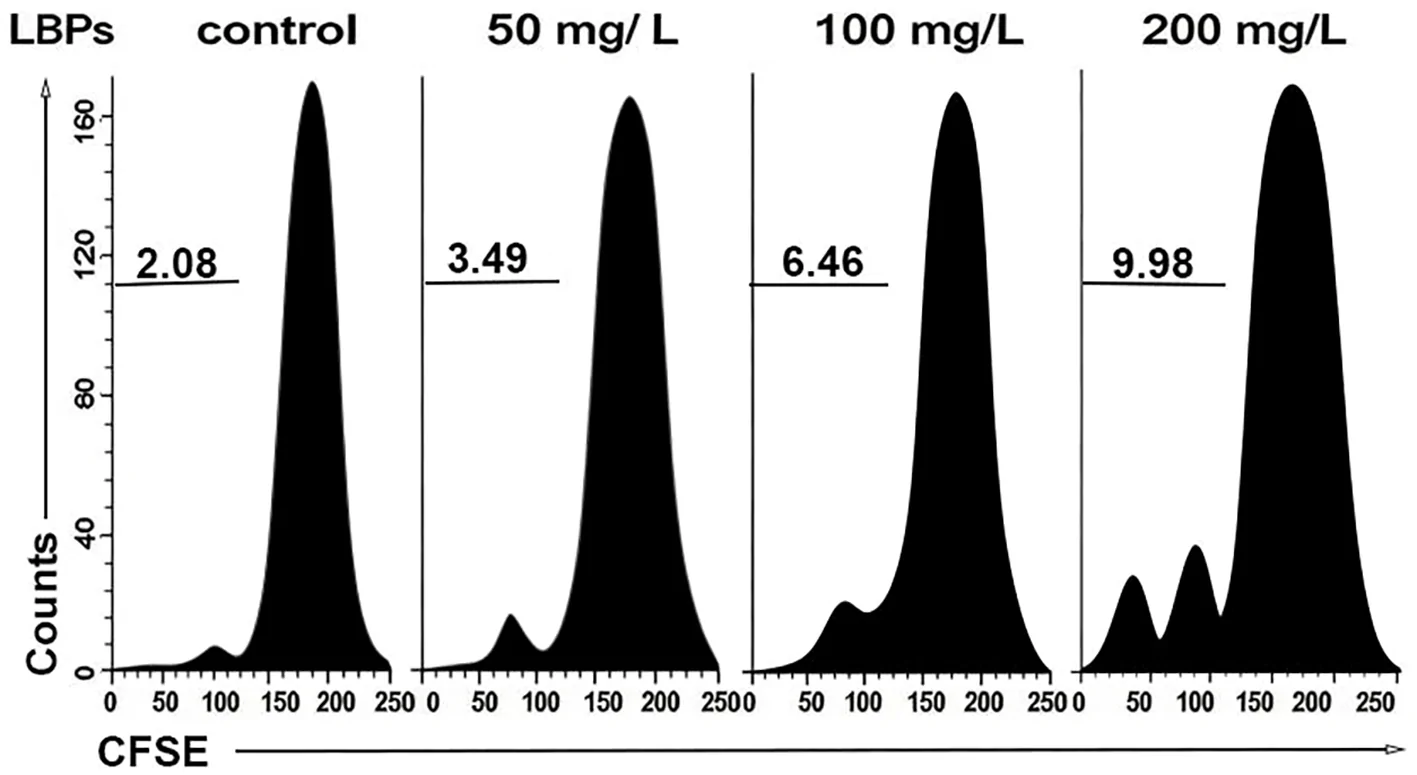
The proliferation of T cells stimulated by DCs with LBPs at different concentrations. The proliferation of allogeneic T lymphocytes in the MLR. LPS-only treatment as control.

**Table 2:** The proliferation of T cells stimulated by DCs with LBPs at different concentrations (n=8, **p < 0.01 compared with the control group).

LBPs (mg/L)	Immature DCs	LBPs-only	LPS-only	50	100	200
Proliferation index	1.93 ± 0.43	2.04 ± 0.32	2.23 ± 0.23	3.27 ± 0.30	6.32 ± 0.52**	10.88 ± 0.98**

### LBPs enhance DC chemotaxis via CCR7 upregulation

Transwell migration assays revealed that DCs treated with 200 mg/L LBPs displayed a significantly enhanced migratory response toward the chemokine MIP-3β compared with untreated controls (**Figure 4A**). This LBPs-induced migration was markedly attenuated by preincubation with an anti-CCR7 blocking antibody (**Figure 4B**), indicating a CCR7-dependent mechanism. Accordingly, CCR7 expression was significantly upregulated by LBPs treatment at both the mRNA (**Figure 4C**) and protein levels (**Figure 4D**) in a dose-dependent manner. Notably, blockade of IL-10 signaling further enhanced LBPs-induced CCR7 expression, albeit modestly (**Figure 4E**), and concomitantly increased the migratory capacity of DCs toward MIP-3β (**Figure 4F**).

**Figure 4:**
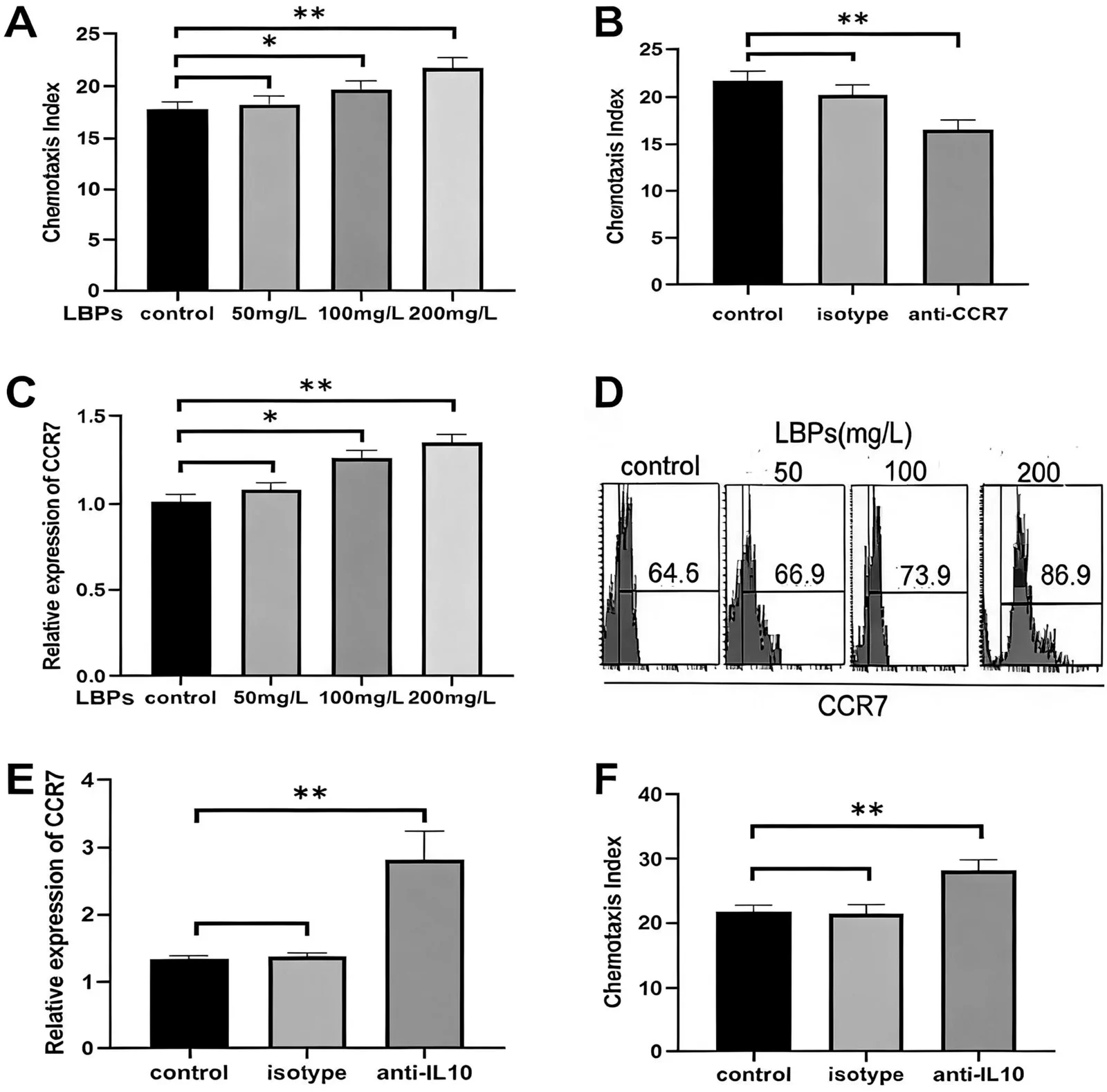
LBPs enhance DC migration via CCR7. **(A)** Migration index of DCs treated with LBPs (0–200 mg/L). **(B)** Anti-CCR7 antibody blocks LBPs-induced migration. **(C, D)** LBPs upregulate CCR7 mRNA **(C)** and protein **(D)**. **(E, F)** IL-10 blockade further increases CCR7 expression **(E)** and DC migration **(F)**. All data are shown as mean ± SD (n = 8 independent biological replicates). *p < 0.05, **p < 0.01 compared with the control group.

## Discussion

Our study demonstrates that LBPs effectively promote the functional maturation and migration of human monocyte-derived DCs. The key findings include the induction of SOCS-1 gene methylation, downregulation of SOCS-1 expression, upregulation of surface maturation markers and IL-12 secretion, inhibition of IL-10 production, enhanced T cell stimulatory capacity, and increased CCR7 expression and chemotaxis toward MIP-3β.

SOCS-1 is a well-established negative regulator of cytokine signaling and DC function (7, 25). Our observation that LBPs induce SOCS-1 methylation and suppress its expression aligns with strategies to enhance DC immunogenicity by inhibiting SOCS-1 (8, 26–28). We investigated the expression of key DNA methyltransferases (DNMT3A and DNMT3B) using real-time PCR. Our results show that LBPs treatment significantly upregulated the mRNA expression of DNMT3A and DNMT3B in a dose-dependent manner. These data suggest that LBPs may promote the methylation of the SOCS-1 promoter by upregulating the expression of these DNMTs. This epigenetic modulation may represent a novel mechanism by which LBPs alleviate the brake on DC activation.

The promoted maturation was evidenced by increased co-stimulatory molecule expression and a shift toward a pro-inflammatory cytokine profile (elevated IL-12, reduced IL-10), which is crucial for driving Th1-type immune responses (29, 30). The enhanced ability of LBPs-treated DCs to stimulate allogeneic T cell proliferation further confirms their improved immunostimulatory function. We have examined the JAK/STAT signaling pathway. Western blot analysis revealed that LBPs treatment resulted in a marked increase in the phosphorylation of JAK2, STAT3, and STAT5, which was consistent with the downregulation of SOCS-1 expression. These findings establish a direct mechanistic link between SOCS-1 methylation, activation of the JAK/STAT signaling pathway, and the altered cytokine-secretion profile (elevated IL-12 and reduced IL-10).

Furthermore, successful migration to lymph nodes is indispensable for DCs to prime naïve T cells. The upregulation of CCR7 and enhanced chemotaxis toward its ligand MIP-3β are hallmarks of mature, migratory DCs (10, 31–35). Our results show that LBPs significantly boost this aspect of DC function. The concurrent reduction in IL-10, a cytokine known to inhibit CCR7 expression and DC migration (36, 37), may contribute to this pro-migratory effect. Our study from the anti-IL-10 neutralizing antibody experiment get the same results. Blocking IL-10 signaling in the presence of LBPs led to a further, albeit modest, increase in CCR7 expression, supporting the concept that LBPs-induced reduction of IL-10 contributes to enhanced CCR7 expression and subsequent migration.

However, the present study is limited by its exclusive reliance on *in vitro* data, and the *in vivo* relevance of our findings—particularly regarding DC migration to lymph nodes and SOCS-1 methylation under pathological conditions—remains unknown. Future work using murine tumor models is therefore needed to validate whether LBPs-treated DCs retain their maturation and migratory phenotypes in the physiological microenvironment, and to determine whether these effects translate into enhanced antitumor immunity *in vivo*.

In conclusion, this study reveals that LBPs enhance human DC maturation and migration, potentially through epigenetic modulation of SOCS-1 and regulation of key functional molecules. These findings provide a mechanistic basis for the immunomodulatory properties of LBPs and suggest their potential as a natural adjuvant in DC-based cancer immunotherapy strategies. Further *in vivo* studies are warranted to validate these effects and explore their therapeutic applications.

## Data Availability

The raw data supporting the conclusions of this article will be made available by the authors, without undue reservation.
